# **Author's Reply**

**DOI:** 10.1371/journal.pgen.0020034

**Published:** 2006-03-31

**Authors:** Matt Kaeberlein, Di Hu, Emily O. Kerr, Mitsuhiro Tsuchiya, Eric A. Westman, Nick Dang, Stanley Fields, Brian K. Kennedy

As in our prior work showing that life span extension by calorie restriction (CR) occurs independently of Sir2 [1], we have attempted to examine the putative role for respiration in life span extension in as comprehensive a manner as possible [2]. We have done this by measuring the effect of CR on life span across a range of glucose concentrations and by comparing the data derived from two different strain backgrounds [2]. We believe it is important to optimize life span extension by CR, in both wild-type and mutant backgrounds, in order to interpret genetic experiments involving CR. In contrast, Lin and colleagues have exclusively used 0.5% glucose for their CR experiments [3–5]. This was the case in their prior work [3], where the response of *cyt1*Δ cells to CR was tested using only one glucose concentration and one strain background. As we show [2], life span extension in the *cyt1*Δ mutant is not maximized at the level of restriction used by Lin et al. [3]. Thus, we speculated that their failure to test multiple glucose levels caused them to mistakenly report that CR does not increase the life span of *cyt1*Δ cells [3]. 

In their correspondence, Lin and Guarente [7] divert attention from our findings that Sir2 and respiration are not required for life span extension by CR in two ways. First, they misinterpret our data in the context of prior data from Kaeberlein et al. [6] showing that both 0.5% and 0.05% glucose increase life span to a similar extent in wild-type PSY316 cells. In fact, our data confirm the findings of Kaeberlein et al. [6]; however, these data are restricted to the wild-type case, and, as we demonstrate [2], it cannot be assumed that the optimal level of restriction will remain constant in various mutant backgrounds. Second, Lin and Guarente [7] suggest that CR at 0.5% glucose might increase life span by a different mechanism than CR at 0.05% glucose, yet they have presented no data to support this hypothesis. In contrast, we have shown that life span extension by CR is independent of Sir2 at either glucose concentration [1], and in our paper [2], we show that respiration is not required for life span extension at either glucose concentration. Since we have reported a statistically significant life span extension from CR at both glucose concentrations in *cyt1*Δ cells, the claims made by Lin and Guarente [7] are untenable. 

In addition to responding to the statements of Lin and Guarente [7], we wish to point out that the single experiment they have chosen to focus on in their correspondence was of relatively minor importance in developing our conclusions. Lin and Guarente [7] do not address the primary finding of our paper [2]: respiration is not required for life span extension by CR. Even neglecting our data for PSY316 *cyt1*Δ cells, we show that CR at either glucose concentration significantly increases the life span of rho^0^ cells, which completely lack mitochondrial DNA [2]. Furthermore, we report here and elsewhere that CR does not result in activation of Sir2, and that Sir2-family proteins are not required for life span extension by CR [1,2,8,9]. In our opinion, these data represent substantial evidence that CR is not mediated by Sir2-family proteins and that increased respiration is not required in life span extension by CR.
